# A multidisciplinary approach to study a couple of monozygotic twins discordant for the chronic fatigue syndrome: a focus on potential salivary biomarkers

**DOI:** 10.1186/1479-5876-11-243

**Published:** 2013-10-02

**Authors:** Federica Ciregia, Laura Giusti, Ylenia Da Valle, Elena Donadio, Arianna Consensi, Camillo Giacomelli, Francesca Sernissi, Pietro Scarpellini, Fabrizio Maggi, Antonio Lucacchini, Laura Bazzichi

**Affiliations:** 1 Department of Pharmacy, University of Pisa, Via Bonanno 6, Pisa, 56126, Italy; 2 Department of clinical and experimental medicine, Division of Rheumatology, University of Pisa, Via Roma 67, Pisa, 56126, Italy; 3 Virology Unit, Pisa University Hospital, Via Roma 67, Pisa, 56126, Italy

**Keywords:** Chronic fatigue syndrome, Whole saliva, Proteomics, Two-dimensional electrophoresis

## Abstract

**Background:**

Chronic Fatigue Syndrome (CFS) is a severe, systemic illness characterized by persistent, debilitating and medically unexplained fatigue. The etiology and pathophysiology of CFS remains obscure, and diagnosis is formulated through the patient’s history and exclusion of other medical causes. Thereby, the availability of biomarkers for CFS could be useful for clinical research. In the present study, we used a proteomic approach to evaluate the global changes in the salivary profile in a couple of monozygotic twins who were discordant for CFS. The aim was to evaluate differences of salivary protein expression in the CFS patient in respect to his healthy twin.

**Methods:**

Saliva samples were submitted to two-dimensional electrophoresis (2DE). The gels were stained with Sypro, and a comparison between CFS subject and the healthy one was performed by the software Progenesis Same Spot including the Analysis of variance (ANOVA test). The proteins spot found with a ≥2-fold spot quantity change and p<0.05 were identified by Nano-liquid chromatography electrospray ionization tandem mass spectrometry. To validate the expression changes found with 2DE of 5 proteins (14-3-3 protein zeta/delta, cyclophilin A, Cystatin-C, Protein S100-A7, and zinc-alpha-2-glycoprotein), we used the western blot analysis. Moreover, proteins differentially expressed were functionally analyzed using the Ingenuity Pathways Analysis software with the aim to determine the predominant canonical pathways and the interaction network involved.

**Results:**

The analysis of the protein profiles allowed us to find 13 proteins with a different expression in CFS in respect to control. Nine spots were up-regulated in CFS and 4 down-regulated. These proteins belong to different functional classes, such as inflammatory response, immune system and metabolism. In particular, as shown by the pathway analysis, the network built with our proteins highlights the involvement of inflammatory response in CFS pathogenesis.

**Conclusions:**

This study shows the presence of differentially expressed proteins in the saliva of the couple of monozygotic twins discordant for CFS, probably related to the disease. Consequently, we believe the proteomic approach could be useful both to define a panel of potential diagnostic biomarkers and to shed new light on the comprehension of the pathogenetic pathways of CFS.

## Background

Chronic Fatigue Syndrome (CFS), also termed Myalgic Encephalomyelitis, is a severe, systemic illness characterized by debilitating, unexplained, persistent fatigue that indeed has wide impact on the quality of life [1-3]. Besides the disabling fatigue, CFS is accompanied by several symptoms that differ among patients: fatigue not improved by rest; headaches; sleep disturbances; difficulties with concentration; memory problems; muscle weakness; musculoskeletal pain, etc. What is more is that all these symptoms may be aggravated by physical or mental activity [4,5]. Therefore, CFS is a medically unexplained disorder diagnosed through symptoms reported by the patient and the exclusion of other medical and psychiatric diseases with the same symptoms. The estimated worldwide prevalence of CFS is 0.4-2.5%, with a peak age of onset between 20 and 40 years, and with a female to male ratio of 3:1 [6].

This clinical disorder is considered a multi-factorial disease with unknown etiology and pathophysiology. Genetic studies have been performed in order to characterize genes that could be useful as CFS biomarkers [7-10]. In particular, it has been proposed that CFS is associated with immunological and inflammatory diseases [7,8]. The over-expression of pro-inflammatory proteins, such as interferon-γ (INF-γ), interleukin-1 (IL-1), and tumor necrosis factor- α (TNF-α), has been reported in CFS [9]. Besides, up-regulated immune-related genes in patients affected by CFS such as lactotransferrin, defensin-α1, integrins [8], CMRF35 antigen, IL-8, HD protein [10], and cathepsin C [9] are supportive of the notion that CFS is characterized by immune system activation. Nevertheless, at present, there are no specific markers of inflammation and/or immune activation which may be useful in the evaluation of patients with CFS, thus making the treatment more complicated.

Viral infection has been usually proposed as a causative agent, but despite the efforts in the elucidation of the role of virus, results are controversial due to a lack of common standard clinical definition and specific biomarkers of the disease [11-16]. Likewise, vaccines have been depicted by some scholars as playing an important role in CFS onset [17,18], while on the other hand, others scholars have rejected this possibility, affirming that the vaccine is safe [19,20].

Whilst in 2011 an International Consensus Panel defined the International Consensus Criteria that identifies the characteristic patterns of the symptom clusters of CFS [21], the diagnosis is formulated through the patient’s history and exclusion of other medical causes and psychiatric problems [5]. Thereby, the availability of biomarkers for CFS could be of great usefulness for clinical research.

Since genetics is presumed to have a role in the etiology of CFS, one area of investigation is the twin studies which could be useful in elucidating the role of genetic and environmental factors in CFS. Nevertheless, a recent study of Byrnes *et al.*[22] on monozygotic twins discordant for CFS, did not identify a biomarker for CFS in the transcriptome of peripheral blood leukocytes supposing that positive findings in prior studies may have resulted from experimental bias. Therefore, we moved to the study of proteins, and, for the first time, we used a proteomic approach to evaluate in CFS the global changes of whole saliva (WS).

Recently, the number of publications related to salivary proteome have significantly increased suggesting human saliva as a biological fluid with an enormous potential to reflect systemic health conditions. Saliva has many advantages in terms of low invasiveness, minimum cost, and easy sample collection and processing [23]. Moreover, saliva has a less complex protein composition than serum or plasma reducing the risk of non specific interactions, and at the same time it represents a useful diagnostic tool since about 30% of blood proteins are also present in saliva. Therefore, human saliva proteomics have proven to be a novel approach in the search for protein biomarkers for detection of diseases [23]. In particular, in the last years, we have obtained encouraging results from the proteomic analysis of human WS in rheumatic diseases [24-28]. Hence, with the present study, we investigated the proteomics salivary profile in a couple of monozygotic discordant for CFS. Differences of salivary protein expression in the CFS patient in respect to the healthy co-twin could be strictly correlated to the disease itself if twins differ only regarding the presence of CFS.

## Methods

### Study design

**Figure 1:**
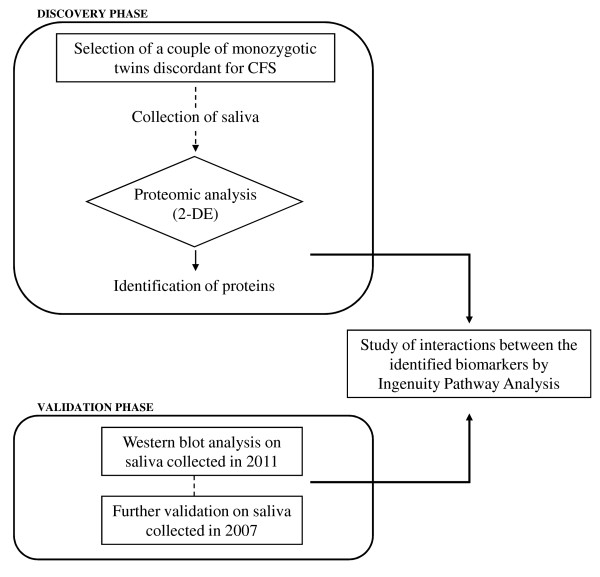
**Study design.** This study adopted a phase approach. The first “discovery” phase characterized the salivary proteomic profile of a CFS. The second “validation” phase assessed the differences of these candidate biomarkers in CFS in respect to healthy subject. Finally, we investigated the biological function of the significantly changing proteins, applying the Ingenuity Pathway Analysis Knowledge base.

This study adopted a phase approach (Figure 1). The first “discovery” phase was aimed at characterising the salivary proteomic profile of a CFS subject in comparison to his healthy twin brother. In the second “validation” phase we performed western blot to assess the ability of these candidate biomarkers to differentiate the CFS from the healthy subject. Finally, we investigated the biological function of the significantly changing proteins, applying the Ingenuity Pathway Analysis (IPA) Knowledge base (Ingenuity® System Inc., Redwood City, CA, USA). This platform enabled us to visualize the potential interactions between the identified biomarker signatures in saliva and other molecules of interest, and to identify biological pathways underlying the CFS disease process.

### Patients

The twins were male, non-smokers, with a body mass index (BMI) of 22.5 (the patient) and 23.5 (the healthy subject) that has not changed over the years. Both brothers are graduated, and practice the same work, in particular they have a business company. They have the same educational background and lifestyle (both are married and have one son). In 2005, when the twins were 31 years old, they both received for the first time an attenuated influenza vaccine. The twins did not have a history for autoimmune disease or other medical conditions. Moreover, there were not any differences in social and other stressors, not even around the time of exposure to the flu vaccine.

Immediately after this vaccine, the patient, who was previously healthy, developed asthenia, progressive weight loss, forgetfulness and concentration difficulty, sleeping disorders, dizziness and fever with indeed determined severe work disability. Physical examination and oral evaluation were negative in both subjects. Questionnaire scores and scales are shown in Table 1. The twins completed an extensive questionnaire regarding demographics, family history, CFS and other health symptoms, social environment, and neuropsychological measures. The patients were classified based on the 1994 Centers for Disease Control and Prevention CFS case definition [3].

**Table 1:** Questionnaire score and scales of CFS patient

	**CFS**	**Healthy twin**
**FIQ**	61,2	4
**VAS pain**	3	0
**VAS fatigue**	8	1
**VAS sleep**	8	2
**FACIT**	26	5
**SF-36**		
*Physical functioning*	50	100
*Role Physical*	0	100
*Bodily Pain*	84	100
*General health status*	10	67
*Vitality*	35	60
*Social activities*	25	100
*Role emotional*	66	100
*Mental health*	64	80

### Questionnaires

**Figure 2:**
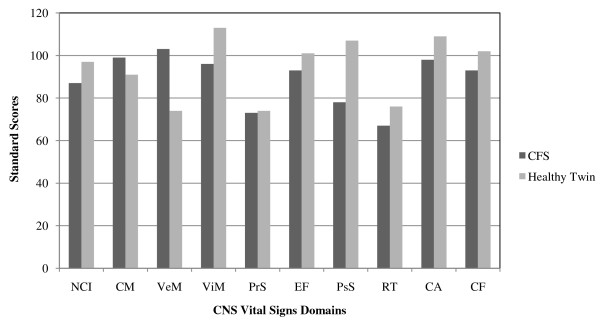
**Results of cognitive function. (NCI)** Neurocognitive index; **(CM)** Composite memory; **(VeM)** Verbal memory; **(ViM)** Visual memory; **(PrS)** Processing speed; **(EF)** Executive function; **(PsS)** Psychomotor speed; **(RT)** Reaction time; **(CA)** Complex attention; **(CF)** Cognitive flexibility.

The twins performed a rheumatologic visit with routine clinical evaluation of medical history. The subjects completed the *Fibromyalgia Impact Questionnaire* (FIQ), in which pain and fatigue severity were assessed with 10-cm visual analogue score scale (VAS) and short form-36 (SF-36). Fatigue was assessed also by means of the *Functional Assessment of Chronic Illness Therapy-Fatigue Scale* (FACIT). We considered the VAS from the FIQ asking if the patient felt rested upon awaking during the last week. Considerable differences were found concerning clinical parameters, in particular fatigue and sleeping disorders. The subjects also completed the CNS Vital Signs©, a computerized neurocognitive assessment platform that enables the objective evaluation and the characterization of the patient’s neurocognitive function. In particular, by standardized methods, the test allows to evaluate the Composite Memory, Verbal memory, Visual Memory, Processing Speed, Executive Function, Psychomotor Speed, Reaction Time, Complex Attention and Cognitive Flexibility. The results of cognitive function are shown in Figure 2. The patient resulted to have more compromised neurocognitive function compared to the healthy brother.

### Laboratory tests

To better classify the subjects the following haematochemical parameters were evaluated:

human leukocyte antigen (HLA) system

immunological parameters: anti nuclear antibodies (ANA), extractable nuclear antigen antibodies (ENA), Rheumathoid Factor (Ra test), anti cardiolipin antibodies (ACLA)

hormone profile: cortisole and adrenocorticotropic hormone (ACTH), serotonin, growth hormone (GH), somatomedin C, progesterone, testosterone, dehydroepiandrosterone (DHEA)

cytokines profile: TNF-α, IL-6, IL-10, IL-2

virological examination performed on blood and pharyngeal swab evaluating herpes virus (HHV), Epstein–Barr virus (EBV), Cocxsakie, parvovirus B19, adenovirus, entero-rhinovirus, influenza and parainfluenza virus.

HLA profile (HLA locus A03, 24; B07, 13; DRB107, 11) and immunological parameters (ENA, Ra test, ACLA were negative, ANA were positive 1:160) were identical in the subjects. A difference in haemotachemical parameters was found concerning TNF-α (18,1 pg/ml and 2,6 pg/ml in patient and healthy twin, respectively) and IL-2 (10 pg/ml and 46 pg/ml in patient and healthy twin, respectively). No difference was found about IL-6, IL-10; and the hormone profiles were normal (no difference was found in twins about cortisole, ACTH, serotonin, GH, somatomedin C, progesterone, testosterone and DHEA). Concerning the virological examination, both subjects had IgG positivity for parvovirus B19, HHV7, HHV6 and varicella (VZV) (Table 2).

**Table 2:** Virological examination

	**CFS**		**Healthy twin**	
	**IgG**	**IgM**	**IgG**	**IgM**
*Ab anti Coxsakie 1-6*	+ type 4	-	-	-
*Ab anti EBV*	-	-	-	-
*Ab anti HSV 1*	+	-	-	-
*Ab anti HSV 2*	+	-	-	-
*Ab anti HHV6*	+	-	+	-
*Ab anti HHV7*	+	-	+	-
*Ab anti VZV*	+	-	+	-
*Ab anti parvo virus B19*	+	-	+	-
*Adenovirus*	-	-	-	-
*InfluenzaType A*	+	-	+	-
*Influenza Type B*	+	-	+	-
*Parainfluenza virus 1 and 2*	-	-	-	-

### Whole saliva collection

Salivary samples were collected from the patient with CFS and from his monozygotic healthy twin brother. Their WS was collected both in 2007 and in 2011 when the twins were 33, and 37 years old, respectively. This means that WS was collected after two and six years from the flu vaccine respectively, simultaneously and at the same interval of time. This study was approved by the local Ethics Committee, and an informed consensus was obtained for diagnostic or clinical purposes.

WS samples were collected early in the morning (between 8 and 11 a.m) according to a standard protocol [28]. The twins were asked to refrain from eating and drinking 2 h before saliva collection. No evidence of oral pathologies or inflammatory processes were observed. The brothers were asked to gently chew a saliva collector sponge (Surescreen Diagnostics LTD; Derby, UK) for 2 minutes, and the saliva collected was immediately centrifuged at 17,000 *g* for 20 minutes at 4°C to yield clear samples. Samples were stored at -80°C. Protein amounts of resulting supernatants were determined using the Bio-Rad DC-protein assay. Bovine serum albumin (BSA) was used as a standard.

### Two-dimensional electrophoresis

Two hundred μg of proteins, for each sample, were filled up to 400 μl in rehydration solution. Immobiline Dry-Strips (GE Health Care Europe; Uppsala, Sweden); 18 cm, linear gradient pH 3–10) were rehydrated overnight in the sample and then transferred to the Ettan IPGphor Cup Loading Manifold (GE Healthcare) for isoelectrofocusing (IEF). IEF was performed at 16°C and the proteins were focused for up to 70000Vh. The second dimension (Sodium Dodecyl Sulphate-Polyacrylamide Gel Electrophoresis; SDS-PAGE) was carried out by transferring the proteins to 12.5% polyacrylamide gel, running at 16 mA/gel and 10°C for about 16 h. The gels were stained with Ruthenium II tris (bathophenanthroline disulfonate) tetrasodium salt (SunaTech Inc.; Suzhou, P. R. China) (RuBP) as described by Aude-Garcia *et al.*[29]. Briefly, after the electrophoresis, the gels were fixed in 1% phosphoric acid (v/v) and 30% ethanol for 1h then were stained overnight with 1 mM RuBP in 1% phosphoric acid and 30% ethanol. After this time the gels were destained for 5 hours in 1% phosphoric acid and 30% ethanol and rinsed in water prior to acquisition by “ImageQuant LAS4010” (GE Health Care). The analysis of images was performed using Progenesis Same Spot (v4.1, Nonlinear Dynamics; Newcastle Upon Tyne, UK) software. This software generates 2DE analyses which are robust and accurate. Briefly, the gels were aligned to place all spots in exactly the same location, and afterwards, the spot detection produced a complete data set since all gels contain the same number of spots, each matched to its corresponding spot on all gels. After 2DE gel alignment and subsequent spot detection, the software calculated background corrected abundance, by determining the lowest intensity value of the image pixels outside the spot’s outlined, and subtracting it from the intensity value of every pixel inside the spot outline. Theses abundances were then normalized compared to a reference gel in order to obtain a normalized intensity value for each spot.

The 2DE experiments were performed in triplicate. The spot volume ratios between the two different conditions were calculated using the average spot normalized volume of the three biological replicates (http://www.nonlinear.com/). The software included statistical analysis calculations.

### 2DE statistical analysis

A comparison between CFS and healthy subject was performed. The significance of the differences of normalized volume for each spot was calculated by the software Progenesis Same Spot including the Analysis of variance (ANOVA test). The protein spots with a ≥2-fold spot quantity change and p<0.05 were cut out from the gel and identified by Nano-liquid chromatography electrospray ionization tandem mass spectrometry (NanoLC-ESI-MS/MS) analysis.

### NanoLC-ESI-MS/MS Analysis by LTQ-Orbitrap Velos analysis

The gel pieces were destained in 100% ethanol during 2 hours. Subsequently, they were rehydrated with 100 μl of 50 mM ammonium bicarbonate for 15 min and dehydrated with 100 μl of 50 mM ammonium bicarbonate in 30% acetonitrile (AcN) for 15 min.

The gel pieces were then dried for 30 minutes in a Centrivap vacuum centrifuge (Labconco, Kansas City, USA). The dried pieces of gel were rehydrated for 45 min at 4°C in 20 μl of trypsin porcine (Sigma-Aldrich; MO, USA) solution (6.25 ng/μl in 50 mM ammonium bicarbonate) and then incubated at 37°C overnight. Extraction of the peptides was performed with 20 μl of 1% trifluoroacetic acid (TFA) for 30 min at room temperature with occasional shaking. The TFA solution containing the proteins was transferred to a polypropylene tube. A second extraction of the peptides was performed with 20 μl of 0.1% TFA in 50% AcN for 30 min at room temperature with occasional shaking. The second TFA solution was pooled with the first one. The volume of the pooled extracts were dried completely and finally resuspended in AcN/formic acid 50%/0.1%.

LC-ESI-MS/MS was performed on a linear trap quadrupole (LTQ) Orbitrap Velos from Thermo Electron (San Jose, CA, USA) equipped with a NanoAcquity system from Waters. Peptides were trapped on a home-made 5 μm 200 Å Magic C18 AQ (Michrom) 0.1 × 20 mm pre-column and separated on a home-made 5 μm 100 Å Magic C18 AQ (Michrom) 0.75 × 150 mm column with a gravity-pulled emitter. The analytical separation was run for 23 min using a gradient of H_2_O/formic acid 99.9%/0.1% (solvent A) and AcN/formic acid 99.9%/0.1% (solvent B). The gradient was run as follows: 0–5 min 95% A and 5% B, then to 65% A and 35% B at 6 min, and 20% A and 80% B at 7 min at a flow rate of 220 nL/min. For MS survey scans, the orbitrap (OT) resolution was set to 60000 and the ion population was set to 5 × 105 with an m/z window from 400 to 2000. For protein identification, up to five precursor ions were selected for collision-induced dissociation (CID). For MS/MS in the LTQ, the ion population was set to 1 × 10e4 (isolation width of 2 m/z), while as for MS/MS detection in the OT, it was set to 1 × 10e5 (isolation width of 2 m/z). The normalized collision energies were set to 35% for CID.

### Protein identification

Peak lists were generated from raw orbitrap data using the embedded software from the instrument vendor (extract_MSN.exe). The monoisotopic masses of the selected precursor ions were corrected using an in-house written Perl script [30]. The peaklist files were searched against the UniProtKB/Swiss-Prot database (Release-2011_08 of 21-Sep-2011) using Mascot (Matrix Sciences, London, UK). Human taxonomy (20323 sequences) was specified for database searching. The parent ion tolerance was set to 10 ppm. Variable amino acid modifications were oxidized methionine and fixed amino acid modifications were carbamidomethyl cysteins. Trypsin was selected as the enzyme, with one potential missed cleavage, and the normal cleavage mode was used. The mascot search was validated using Scaffold 3.6.0 (Proteome Software, Portland, OR). Only proteins matching with two different peptides with a minimum probability score of 95% were considered identified.

### Western blot analysis

To validate the expression changes found with 2DE, we used western blot analysis. Mono-dimensional western blot was performed as previously described [31] for 14-3-3 protein zeta/delta, cyclophilin A (CYPA), Cystatin-C, and Protein S100-A7. Briefly, aliquots of WS samples were run on 12% SDS-PAGE gels (14-3-3 protein zeta/delta, CYPA, Cystatin-C) or on 15% SDS-PAGE gels (Protein S100-A7). The amount of proteins loaded and the dilution of primary antibody was different depending on each protein analysed (14-3-3 protein zeta/delta and CYPA: 50 μg of proteins and dilution of 1:500, Cystatin-C 20 μg and a dilution of 1:200, Protein S100-A7 40 μg and 1:200). The immunocomplexes were detected using a peroxidase-labelled secondary antibody (goat anti-rabbit, 1:10000 dilution; goat anti-mouse, 1:20000; donkey anti-goat, 1:5000). Immunoblots were developed using the ECL detection system. The chemiluminescent images were acquired by LAS4010 (GE Health Care).

To visualize the different expression of zinc-alpha-2-glycoprotein (ZAG), aliquots of WS corresponding to 300 μg of proteins, were separated by 2DE using 3–10 linear strips (18 cm) and 12,5% gels before western blot analysis with specific antibody (1:1000 dilution).

### Western blot statistical analysis

The antigen-specific bands were quantified using the Image Quant-L (GE Health Care) software. Data were reported as optical density and subjected to statistical analysis with Student t-test making a comparison of protein expression levels between CFS and control.

### Signaling pathway analysis

Proteins differentially expressed, were functionally analyzed using the Ingenuity Pathways Analysis (IPA) software v14400082 (2012 Ingenuity Systems, Inc. http://www.ingenuity.com) with the aim to determine the predominant canonical pathways and interaction network involved. Swiss-Prot accession numbers and official gene symbols were inserted into the software along with corresponding comparison ratios between the CFS patient and the healthy brother. The network proteins associated with biological functions and/or diseases in the Ingenuity Pathways Knowledge Base were considered for the analysis. These networks are scored for degree of relevance with values >3 having a 99.9% confidence level of not being generated by random chance alone. The created genetic networks describe functional relationships among proteins based on known associations in the literature.

## Results

### Proteomic analysis and validation by western blot

**Figure 3:**
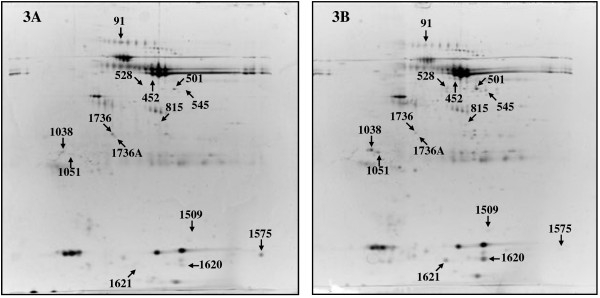
**2DE patterns of human saliva.** 2DE patterns of human WS: healthy subject **(A)** and patient with CFS **(B)**. A total of 200 μg of proteins was separated by 2DE using 18 cm pH 3-10L strips and 12.5% SDS-PAGE. The 13 spots differently expressed in CFS in respect to control are indicated.

Figure 3 illustrates the salivary profiles of the healthy twin brother (A) and of the patient (B). Overall, we found 13 spots differently expressed in CFS in respect to the control with a fold variation ≥ 2. Nine spots were up-regulated in CFS and 4 down-regulated. A list of identified proteins, with molecular weight (MW), isoelectric point (pI), coverage and score values of NanoLC-ESI-MS/MS is given in Table 3. Table 4 shows the statistical analysis: the normalized volume (mean ± SD) of each spot with p-value and fold-change in expression levels between samples.

**Table 3:** MS/MS data of salivary proteins found differentially expressed between CFS patient and his healthy twin

**n° spot**	**Protein name**	**ID**	**Gene name**	**MW**		**pI**		**Matched peptides**	**Coverage**	**Best ion score**	**Peptide identified**
				**obs**	**th**	**obs**	**th**				
*Immune system*											
**91**	Polymeric immunoglobulin receptor	**P01833**	**PIGR**	80	83	5.6	5.6	7	12%	82.8	(R)QSSGENcDVVVNTLGKR(A)
**815**	Ig alpha-1 chain C region	**P01876**	**IGHA1**	38	38	6.2	6.1	2	7%	60.9	(K)TFTcTAAYPESK(T)
**1509**	Cyclophilin A	**P62937**	**PPIA**	18	18	7.5	7.7	6	46%	63.1	(K)KITIADcGQLE(-)
*Inflammatory response*											
**1575**	Cystatin-C	**P01034**	**CST3**	16	16	9.3	9	3	25%	49.7	(R)ALDFAVGEYNK(A)
**1620**	Cystatin-B	**P04080**	**CSTB**	14	11	6.6	7	4	70%	51.3	(K)VHVGDEDFVHLR(V)
**1621**	Protein S100-A7	**P31151**	**S100A7**	14	11	6	6	2	23%	25.9	(R)SIIGmIDmFHK(Y)
*Metabolism*											
**545**	6-phosphogluconate dehydrogenase, decarboxylating	**P52209**	**PGD**	48	53	6.7	6.8	2	6%	69.2	(K)GILFVGSGVSGGEEGAR(Y)
**1736**	Zinc-alpha-2-glycoprotein	**P25311**	**AZGP1**	36	34	5.5	5.7	8	27%	67.5	(R)AKAYLEEEcPATLR(K)
**1736A**	Zinc-alpha-2-glycoprotein	**P25311**	**AZGP1**	36	34	5.5	5.7	2	5%	94.3	(R)AKAYLEEEcPATLR(K)
*Signal transduction*											
**528**	Rab GDP dissociation inhibitor beta	**P50395**	**GDI2**	50	51	5.9	6.1	3	9%	50.9	(R)mTGSEFDFEEmKR(K)
**1038**	14-3-3 protein sigma	**P31947**	**SFN**	33	28	4.6	4.7	8	38%	84.7	(K)SNEEGSEEKGPEVR(E)
**1051**	14-3-3 protein zeta/delta	**P63104**	**YWHAZ**	33	28	4.8	4.7	3	20%	45.5	(K)LAEQAERYDDmAAcmK(S)
*Others*											
**452**	Serum albumin	**P02768**	**ALB**	57	69	6.2	5.9	8	14%	84.5	(K)VPQVSTPTLVEVSR(N)
**501**	Alpha-amylase 1	**P04745**	**AMY1A**	52	58	6.5	6.5	5	11%	98.3	(K)TGSGDIENYNDATQVR(D)

(obs: observed; th: theoretical).

*ID*: SwissProt accession number of the protein; *Gene name*: name of the corresponding gene; *Matched peptides*: the number of unique peptides on which the protein identification is based; *Coverage*: the percentage of the protein's sequence represented by the peptides identified by MS; *Best ion score*: it is a measure of how well the observed MS/MS spectrum matches to the stated peptide; *Peptide identified*: each spot corresponding to one certain protein had at least one of the shown peptides identified.

**Table 4:** Results of 2DE statistical analysis

**n° spot**	**Protein name**	**ctrl** ± **SD**	**CFS** ± **SD**	**Fold CFS**	**p-value**
**91**	Polymeric immunoglobulin receptor	2.35e^+06^ ± 3.31e^+05^	1.09e^+06^ ± 1.67e^+05^	- 2.2	0.003
**452**	Serum albumin	1.72e^+05^ ± 1.64e^+04^	7.78e^+05^ ± 9.87e^+04^	+ 4.5	0.0001
**501**	Alpha-amylase 1	1.13e^+05^ ± 3.15e^+04^	5.31e^+05^ ± 6.59e^+04^	+ 4.7	0.0009
**528**	Rab GDP dissociation inhibitor beta	1.27e^+05^ ± 2.27e^+04^	.88e^+05^ ± 6.66e^+04^	+ 3.1	0.001
**545**	6-phosphogluconate dehydrogenase. decarboxylating	5.18e^+05^ ± 7.04e^+04^	1.32e^+06^ ± 3.09e^+04^	+ 2.5	0.01
**815**	Ig alpha-1 chain C region	5.36e^+05^ ± 7.90e^+04^	8.61e^+04^ ± 1.90e^+04^	- 6.2	0.0003
**1038**	14-3-3 protein sigma	5.08e^+05^ ± 7.43e^+04^	1.73e^+06^ ± 1.12e^+05^	+ 3.4	0.0002
**1051**	14-3-3 protein zeta/delta	1.19e^+05^ ± 3.01e^+04^	5.80e^+05^ ± 9.25e^+04^	+ 4.9	0.0009
**1509**	Cyclophilin A	2.64e^+05^ ± 5.54e^+04^	7.00e^+05^ ± 7.87e^+04^	+ 2.6	0.02
**1575**	Cystatin-C	2.47e^+06^ ± 1.13e^+06^	2.55e^+05^ ± 4.38e^+04^	- 9.7	0.001
**1620**	Cystatin-B	3.08e^+06^ ± 3.47e^+05^	8.77e^+06^ ± 4.04e^+05^	+ 2.8	0.01
**1621**	Protein S100-A7	1.46e^+05^ ± 2.34e^+04^	5.80e^+05^ ± 2.25e^+05^	+ 4.0	0.004
**1736/A**	Zinc-alpha-2-glycoprotein	2.37e^+06^ ± 8.06e^+03^	9.61e^+05^ ± 8.30e^+04^	- 2.5	0.008

The normalized volume (mean±SD) of each spot with p-value and fold-change in expression levels in whole saliva of CFS in respect to control.

**Figure 4:**
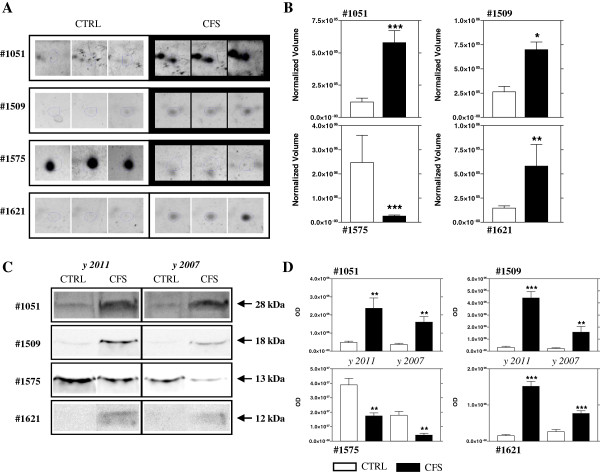
**Proteins differentially expressed in CFS saliva.** Enlarged images of 4 proteins found up- or down-regulated in WS of CFS patient in respect to control subject **(A)**, and histograms of the normalized volume (M±SD) obtained with 2DE analysis for these proteins **(B)**. Western blot analysis of these 4 proteins in WS collected both in 2007 and in 2011 **(C)**, and the respective histograms of the optical density (OD). Each bar represents the mean±SD **(D)**. As depicted in Figure 4D, the modulation illustrated in Figure 4B has been confirmed. (* p< 0.05, ** p< 0.01, *** p< 0.001).

Western blot analysis with specific antibodies was used to validate the expression changes of four proteins: 14-3-3 protein zeta/delta (n°1051), CYPA (n°1509), Cystatin-C (n°1575), and Protein S100-A7 (n°1621) (Figure 4A, 4B). The immunoblot for each protein tested is shown in Figure 4 panel C. For each protein the optical density of specific immunoreactive band was determined and the resulting values compared (Figure 4D). With 2DE we found a significant increase in CFS of 14-3-3 protein zeta/delta, CYPA, and Protein S100-A7 with a p-value of 0.0009, 0.02, 0.004 respectively (Figure 4B) that was confirmed with western blot. Also the decrease of Cystatin-C (p-value=0.001) was confirmed by western blot analysis. These experiments of validation were carried out on WS collected both in 2007 and in 2011, when the twins were 33 and 37, and in both case the results supported those of the 2DE study.

**Figure 5:**
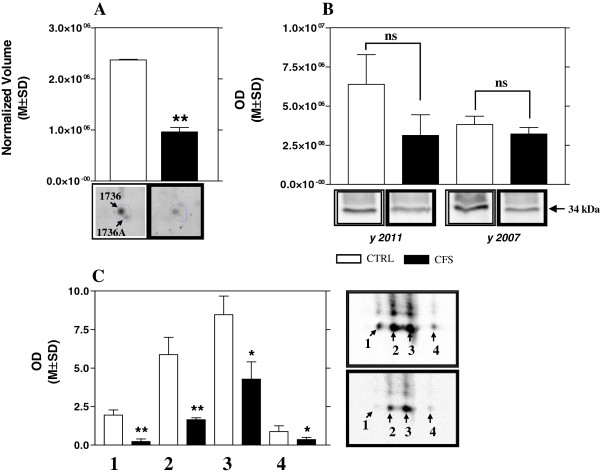
**Expression of ZAG.** Enlarged image of ZAG in 2DE gel with the histogram of the normalized volume (M±SD) **(A)**, and validation of ZAG with mono-dimensional western blot **(B)**, and 2DE western blot **(C)**. With 2DE immunoblotting we found 4 spots corresponding to ZAG (spot 1- spot 4) with pI values ranging from 5.4 (spot 1) to 5.9 (spot 4). The decrease in CFS in respect to control was observed for all spots detected by immunoblotting. (* p< 0.05, ** p< 0.01, *** p< 0.001, ns not significant).

Moreover, we conducted mono-dimensional western blot analysis also for ZAG, but the lower value in the twin with CFS was not significant with a fold variation of 2.0 and 1.2 in WS collected in 2007 and 2011 respectively (Figure 5 panel B). Actually, by 2DE we found two close spots corresponding to ZAG (n°1736, n°1736A) (Figure 5 panel A, and Table 3), and the mono-dimensional electrophoresis could not allow us to appreciate the real difference of this protein in WS. Therefore, we performed 2DE immunoblotting to evaluate the differences of expression observed in different spots. The detected immunoreactive spots were four and they corresponded to pI values ranging from 5.4 (spot 1) to 5.9 (spot 4) (Figure 5 panel C). The significant decrease in CFS in respect to control was observed for all spots detected by 2DE immunoblotting confirming the result obtained by 2DE and NanoLC-ESI-MS/MS.

### Potential Biomarkers found in CFS

Table 5 summarizes the potential biomarkers found in CFS with their functional properties. Moreover, we performed a preliminary validation on a larger number of patients for one of these proteins: CYPA (data not shown). ELISA assay for CYPA was carried out on WS of 20 patients affected by CFS, and 20 healthy subjects, confirming the up-regulation of this protein: the p-value was 0.03, and the fold variation of this protein in CFS respect to controls was 2.4.

**Table 5:** Functional properties of salivary proteins found differentially expressed between CFS patient and his healthy twin

**n°**	**Protein name**	**Functional properties**
*Immune system*		
**1509**	cyclophilin A	Protein with peptidyl-prolyl cis-trans isomerase activity, which accelerates the folding of proteins and catalyzes conformational changes in several cellular processes.
**815**	Ig alpha-1 chain C region	Ig alpha is the major immunoglobulin class in body secretions. It may serve both to defend against local infection and to prevent access of foreign antigens to the general immunologic system.
**91**	Polymeric immunoglobulin receptor	This receptor binds polymeric IgA and IgM at the basolateral surface of epithelial cells. The complex is then transported across the cell to be secreted at the apical surface. During this process a cleavage occurs that separates the extracellular (known as the secretory component) from the transmembrane segment
*Inflammatory response*		
**1621**	Protein S100-A7	It has been associated with increased inflammatory cell infiltrates in breast cancer and various inflammatory disorders, and recently its role in the production of pro-inflammatory molecules has been demonstrated on breast tumor cells.
**1575**	Cystatin-C	Proteins with a role in protein catabolism, in regulation of hormone processing and bone resorption, in inflammation, in antigen presentation and T-cell dependent immune response as well as resistance to various bacterial and viral infections.
**1620**	Cystatin-B	Cystatin-B is an intracellular thiol proteinase inhibitor. Tightly binding reversible inhibitor of cathepsins L, H and B.
*Signal transduction*		
**1051**	14-3-3 protein zeta/delta	Phosphoserine/phosphothreonine-recognition proteins implicated in the regulation of many intracellular signaling. 14-3-3 proteins form homo- or hetero dimers and they can bind different molecules (e.g. kinases, phosphatases, transmembrane receptors and transcription factors).
*Metabolism*		
**1736**	Zinc-alpha-2-glycoprotein (ZAG)	It is mainly known as an adipokine responsible for lipid degradation that causes loss of adipose tissue in cancer cachexia. Beyond this action, a role of ZAG in the activation of AMP kinase, an important regulator of energy metabolism, in human skeletalmuscle cells has emerged.

### Pathway and network analysis

**Figure 6:**
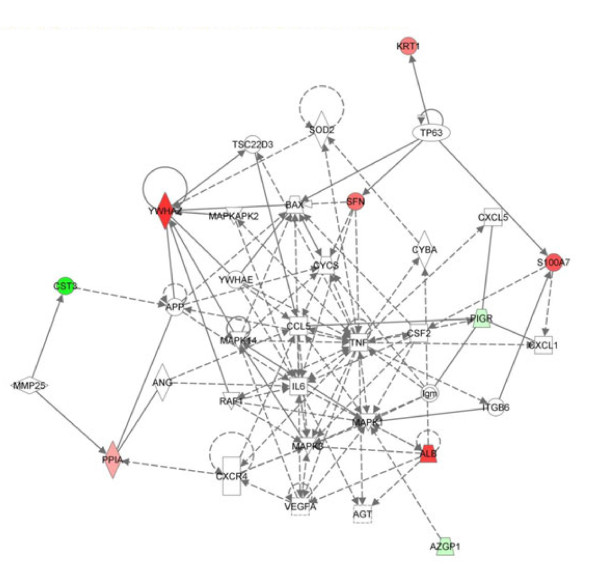
**Network analysis of differentially expressed proteins.** The canonical pathway of differentially expressed proteins with the highest score, obtained by using IPA. Proteins increased in CFS are colored in red and those that decrease in abundance relative to healthy control are colored in green.

The 13 proteins that distinguish the WS of the CFS patient from the healthy subject were used for bioinformatic analysis to identify biological function and pathways involved in CFS using IPA. IPA software allows to investigate the relationship between those proteins and various biological pathways. Figure 6 shows the predominant canonical pathways generated by uploading proteins in Table 4. The software generated different networks. The one with the highest score (score value 21) encompassed 35 proteins and was associated with “Cellular movement, Hematological System Development and Function, Immune Cell Trafficking”. Eight of these proteins were found in our analysis. The top function linked with these biomarkers was the inflammatory response (p-value 3.78E^-05^-4.99E^-02^) that is associated with 8 of our proteins.

## Discussion

This work, for the first time, used a proteomic approach to evaluate the global changes in WS in a patient with CFS. In our large cohort of patients with CFS, we selected a patient with a monozygotic twin brother; despite the two subjects had the same genetic, education, lifestyle and practiced the same work, one of them developed CFS after a vaccine. Despite this pilot study analysed only two subjects, the differences of salivary protein expression could be linked to the disease itself, since the twins differed only regarding the presence of CFS. By this way, our study on a couple of twins could permit the focusing on some biomarkers representing the prelude to a targeted search for these proteins in a wide number of patients, in order to confirm their usefulness in the diagnosis and therapy of CFS. At present, few proteomics works on CFS have been carried out on cerebrospinal fluid [32,33], and on serum [34] while in our study we decided to explore the WS of CFS patient preferring to adopt a less invasive procedure in collecting the samples. Although WS is a less complex fluid, it has been proved to be a promising diagnostic tool [23-28], and we found an alteration of protein patterns in the patient in respect to his control.

### Immune system

Concerning the proteomic analysis on WS, our results showed an increase of CYPA with a fold of 2.6, which was also confirmed by the western blot analysis and verified in WS collected in different years. CYPA was previously found up-regulated in WS of patients affected by Sjögren’s syndrome and Systemic sclerosis [28,35] sustaining the role of CYPA in the pathogenesis of immune-mediated disorders [36]. Moreover, viral infections have been discovered to promote the secretion of CYPA [37-39] supporting the hypothesis that a persistent viral infection may contribute to the pathogenesis of CFS [11].

Besides, the decrease of Ig alpha-1 chain C and of polymeric immunoglobulin receptor (pIgR) that we observed could have promoted the viral infection/immunological alteration. In fact, Ig alpha-1 chain C has a role in preventing access of foreign antigens to the general immunologic system. PIgR ensures humoral defense in the mucosa against incoming pathogens [40] and it is responsible for intracellular neutralization of some viruses through its secretory component [41]. For example, Takiguchi and colleagues observed a reduction in pIgR in dextran sodium sulfate-induced colitis (DSS) [42] supporting the preventive role of pIgR proposed by Murthy *et al.*[43]. DSS is a model that has been widely used to investigate the mechanisms of inflammatory bowel diseases, which are chronic inflammatory disorders [42]. The mucosal immune system is primarily responsible for preventing infection caused by luminal microorganisms by inhibiting bacterial attachment or invasion, and it has been proposed that pIgR may be necessary for maintaining the basal tone of innate immunity in the intestinal environment [42,43]. Therefore, these studies support the involvement of pIgR in the protection against environmental antigens [42,43].

Furthermore, the altered regulation of the immune system in CFS patients is one of the area of interest in the research of biomarkers for CFS. Brenu and colleagues found immunological abnormalities, in particular a reduced cytotoxic activity of innate immune cells, which they proposed as a potential diagnostic tool for CFS [44,45].

In this direction the increase of Human S100A7 (psoriasin) that is overexpressed in inflammatory diseases could be also seen.

### Inflammatory response

Psoriasin has been associated with increased inflammatory cell infiltrates in breast cancer and various inflammatory disorders [46-49]. As shown by the pathway analysis, the network built with our proteins highlights the involvement of inflammatory response in CFS pathogenesis.

Among the proteins changed in CFS in respect to the healthy subject, we found two proteins which belong to the cysteine proteinase inhibitors family: cystatin C and B. Cystatins function as tight-binding inhibitors of cathepsins, which are cysteine proteinases involved in a number of important cellular processes including inflammation [50]. A broad spectrum of biological roles have been suggested for cystatins, including a role in protein catabolism, in the regulation of hormone processing and bone resorption, in inflammation, in antigen presentation and T-cell dependent immune response as well as resistance to various bacterial and viral infections [50]. Salivary cystatins are likely to exert certain antiviral effect as they can interfere with events in viral replication [47-52]. Our results showed an up-regulation of cystatin B, and a down-regulation of cystatin C. These two cystatins belong to two different subtypes of cystatin, type I and II respectively. It was demonstrated that they have different functions, e.g., type I cystatins are up-regulated in tumor tissue while type II cystatins are generally down-regulated in tumors [50]. Therefore, we believe our findings could suggest that the balance between cysteine proteinases and their inhibitors is impaired in CFS.

### Signal transduction

In the WS of our CFS patient, we detected the significant increase of two members of 14-3-3 family in respect to his healthy brother. This family is a class of phosphoserine/phosphothreonine (pSer/Thr)-recognition proteins implicated in the regulation of many intracellular signaling [53]. Many organisms express multiple isoforms, and we found isoforms sigma and zeta/delta. Therefore, 14-3-3 proteins are involved in a wide range of pathological processes [53] and by means, the increase is probably not CFS-specific. For example, we previously found an up-regulation of 14-3-3 proteins also in rheumatoid arthritis [26]. In addition, Matsuo *et al.* have recently found 14-3-3 protein level increased in a patient with Sjögren’s syndrome, but the patient was originally suspected to suffer from Creutzfeldt-Jakob disease (CJD) [54] since 14-3-3 protein was considered an important diagnostic marker of CJD [55]. So they supported the assumption that 14-3-3 is non-specifically increased in various diseases.

### Metabolism

Finally, we observed a down-regulation in CFS of ZAG. We found two spots in 2DE gels corresponding to this protein and, since the specific antibody has a greater sensitivity in respect to any staining protocol, with western blot we found two other spots, all confirming the decrease. ZAG is mainly known as an adipokine responsible for lipid degradation that causes loss of adipose tissue in cancer cachexia. However, beyond this action, a role of ZAG in the activation of AMP kinase (AMPK), an important regulator of energy metabolism, in human skeletalmuscle cells has emerged [56]. The mechanism may be involved in mediating the effects of ZAG in relation to increased energy utilization. This is interesting if we consider that several studies suggest an organic cause for CFS related to defects in oxidative metabolism. In particular, individuals with CFS reach exhaustion (in the presence of reduced sarcoplasmic ATP concentrations) much more rapidly in respect to healthy subjects with resultant acceleration of glycolysis in working skeletal muscles [57]. Moreover, one of the primary symptoms of CFS is muscle pain and weakness; this process is probably the result of cellular changes [57] such as reduction in the number of motor units and atrophy due to disuse. Considering that Russell and colleagues have demonstrated the ability of ZAG to reduce reactive oxygen species (ROS) production and to counter muscle atrophy associated with insulin resistance and other catabolic conditions [58], the decrease of ZAG that we found in WS seems to support the hypothetical role of oxidative stress in CFS. From the same point of view we can explain the increase of 6-phosphogluconate dehydrogenase in WS of CFS. This is an enzyme of the oxidoreductase class that allows the production of NADPH which is necessary for protection against ROS.

## Conclusions

In conclusion, this study on a couple of monozygotic twins discordant for CFS point out some proteins which are useful both to define a panel of potential diagnostic biomarkers and to shed new light on the comprehension of the pathogenetic pathways of CFS. There is a clear need to extend our results on a larger number of subjects with similar medical history, and our preliminary results are encouraging for our future study.

## Competing interests

The authors declare that they have no competing interests.

## Authors’ contributions

FC, LG and AL designed the study, coordinated the research, analyzed data and wrote the manuscript; FC, YDV and ED carried out proteomic analysis; AC and PS carried out the neurocognitive tests; CG and FS carried out the clinical biochemical analysis; FM carried out the virological examination; LB carried out the clinical evaluation of the patient, participated in the design and coordination of study and helped to draft the manuscript. All authors read and approved the final manuscript.
